# Successful Management of Insulin Allergy and Autoimmune Polyendocrine Syndrome Type 4 with Desensitization Therapy and Glucocorticoid Treatment: A Case Report and Review of the Literature

**DOI:** 10.1155/2014/394754

**Published:** 2014-11-19

**Authors:** Joselyn Rojas, Marjorie Villalobos, María Sofía Martínez, Mervin Chávez-Castillo, Wheeler Torres, José Carlos Mejías, Edgar Miquilena, Valmore Bermúdez

**Affiliations:** ^1^Endocrine and Metabolic Diseases Research Center, School of Medicine The University of Zulia, Maracaibo 4004, Venezuela; ^2^Endocrinology Department, Maracaibo University Hospital (SAHUM), Maracaibo 4004, Venezuela

## Abstract

*Introduction*. Insulin allergy is a rare complication of insulin therapy, especially in type 1 diabetes mellitus (T1DM). Key manifestations are hypersensitivity-related symptoms and poor metabolic control. T1DM, as well as insulin allergy, may develop in the context of autoimmune polyendocrine syndrome (APS), further complicating management. *Case Report*. A 17-year-old male patient, diagnosed with T1DM, was treated with various insulin therapy schemes over several months, which resulted in recurrent anaphylactoid reactions and poor glycemic control, after which he was referred to our Endocrinology and Immunology Department. A prick test was carried out for all commercially available insulin presentations and another insulin scheme was designed but proved unsuccessful. A desensitization protocol was started with Glargine alongside administration of Prednisone, which successfully induced tolerance. Observation of skin lesions typical of vitiligo prompted laboratory workup for other autoimmune disorders, which returned positive for autoimmune gastritis/pernicious anemia. These findings are compatible with APS type 4. * Discussion*. To our knowledge, this is the first documented case of insulin allergy in type 4 APS, as well as this particular combination in APS. Etiopathogenic components shared by insulin allergy and APS beg for further research in immunogenetics to further comprehend pathophysiologic aspects of these diseases.

## 1. Introduction

Type 1 diabetes mellitus (T1DM) is an autoimmune disease where adaptive immunity-mediated destruction of pancreatic *β* cells leads to an absolute absence of insulin [1]. Infiltration of Langerhans islets by mononuclear cells, as well as antipancreatic islet antibodies and circulating islet-reactive T cells, is prominent features of this pathology [2]. Notwithstanding the key role played by genetic susceptibility for the development of T1DM, environmental and nongenetic factors are an equally relevant component in the pathophysiology of this disease [3]. Due to the irreversibility of the damage to pancreatic *β* cells, patients with T1DM require lifetime insulin therapy to preserve adequate metabolic control [4]. In these subjects, hypersensitivity to insulin is a rare condition, yet it represents a substantial challenge for attending physicians, since in these cases, the predominant immune component attacks not only the pancreatic islet, but also insulin itself, the main therapeutic measure for the disease.

**Figure 1:**
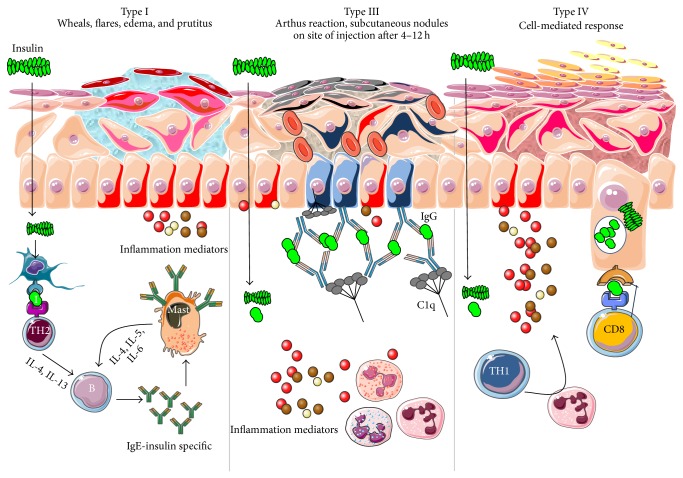
Types of hypersensitivity associated with insulin-related allergy reactions. Type I hypersensitivity reaction is characterized to be a TH2-controlled IgE-insulin specific mediated process, with local edema, itching, wheals, and flares, which could also be associated with angioedema. Type III hypersensitivity is mediated by antigen-antibody complex and recruitment of complement C1q, with subsequent edema, necrosis, and nodule formation. Finally, Type IV reactions are CD8-cytotoxic specific with subcutaneous edema, itching, and hyperkeratosis.

In early documented cases of insulin allergy, hypersensitivity was developed in response to administration of animal-origin insulins, which possess a great antigenic potential due to alterations in their structure, presence of contaminants, and highly immunogenic components such as C-peptide and proinsulin [5–7]. Moreover, IgG anti-insulin antibodies can induce a primary form of insulin resistance, progressive dysglycemia, and lipoatrophy [5]. However, the introduction of human recombinant insulins in the early 1980s has led to a drastic decrease in incidence of these cases, currently estimated at <1% to 2.4% [8]. Clinically, manifestations range from local reactions at the site of injection to severe cases of potentially life-threatening generalized anaphylaxis, and the disease has been described as type I hypersensitivity (IgE-mediated) [9], type III (immune complex-mediated) [10], and type IV delayed hypersensitivity to components added to insulin preparations such as cresol, protamine, and epoxy resin [7, 8, 10–25]; see Figure 1. Tendency to developing hypersensitivity towards insulin relies on its structural modifications in comparison to endogenous insulin, which would modify central tolerance for T lymphocytes [10–25] (Table 1). Management of such cases involves a change of insulin presentation, but, ultimately, most individuals are set through a desensitization protocol [7, 26].

It has been observed that T1DM can coexist with other autoimmune diseases, including vitiligo. The latter is an autoimmune skin disorder characterized by loss of pigmentation due to melanocyte destruction [27]. Several genetic factors have been involved in its pathogenesis, including polymorphisms in HLA-DRB1∗07:01, HLA-B∗44:03, HLA-A∗02:01 y HLA-A∗33:01 [27], and other intrinsic defects in melanocytes, which affect its ability to sustain UV stress [28]. Cellular immunity has been proved to participate in this scenario, along with autoreactive CD8+ T cells which are the effector cells in this disease [29]. The combination of T1DM and vitiligo can be seen as part of the autoimmune polyendocrine syndromes (APS), alongside various other glandular autoimmune dysfunctions [30], with a prevalence of 2–10% [16]. Classification of entities in the APS spectrum is complex and is based on the distinct combinations of autoimmunity-targeted organs [31]; see Table 2. In this broad spectrum of autoimmune diseases, T1DM patients can also be diagnosed with autoimmune thyroid disease (~30%), celiac disease (4–9%), autoimmune gastritis/pernicious anemia (5–10%), and Addison's disease (~0.5%) [30, 31].

The following case concerns a teenage boy with T1DM, vitiligo, and autoimmune gastritis, presenting with a severe case of allergy to multiple insulins, managed with a desensitization protocol.

## 2. Case Presentation

A 17-year-old teenager patient, from Los Puertos de Altagracia community (Miranda municipality, Zulia state), who was diagnosed with T1DM on October 2012 after a hyperglycemic crisis complicated with diabetic ketoacidosis, treated only with fast-acting insulin (Crystalline) due to type I hypersensitivity to intermediate-lasting insulin NPH (Neutral Protamine Hagedorn), and long-lasting insulin Glargine (Lantus). He was referred to the Endocrinology and Immunology outpatient unit at our institute due to persistent bilious emesis, abdominal pain, weight loss, muscular wasting, and daily sprouts of wheals in arms, legs, and torso, usually 30 minutes after the injection of insulin. The following findings were described after written consent was obtained from his mother—his current legal guardian.

**Figure 2:**
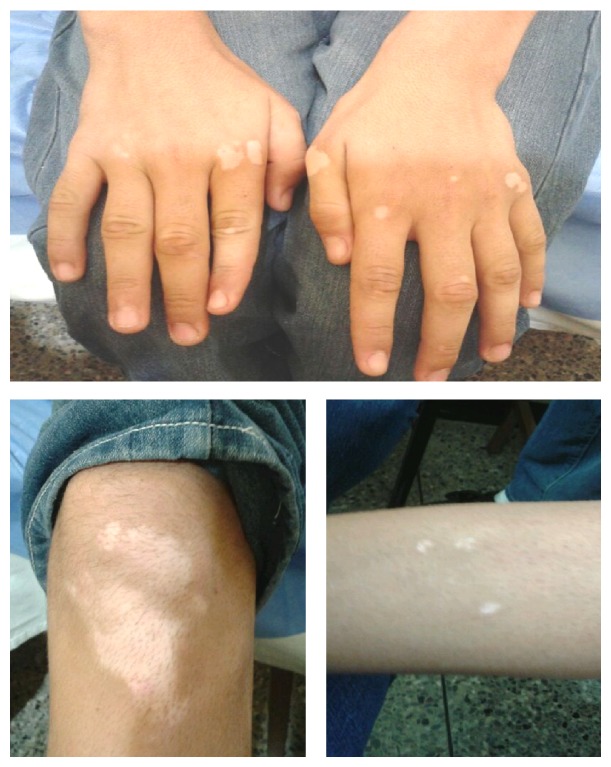
Hypochromic skin lesions associated with vitiligo.

This child was the result of an uneventful pregnancy. At birth, imperforate anus was diagnosed and corrected before 3 months of life. During his infancy, he achieved all stages of neurological development satisfactorily. His height—1.83 m—is a noteworthy trait since parents do not exceed 1.70 m in this regard. Earliest manifestations of vitiligo were observed at 12 years of age, with symmetrical patches of hypopigmented skin in ankles, knees, and hands (Figure 2). His family history includes a younger brother who was born with transient hypoglycemia, low birth weight, and multiform erythema and uncle from his mother side with vitiligo.

His diabetic debut was further investigated and the mother was reinterrogated. During his first hyperglycemic crisis—October 2012—he was brought to the ER with intense tiredness, weight loss, polyuria, polydipsia, abdominal pain, emesis, and glucose levels >400 mg/mL. He was treated with fluids and one Crystalline insulin 10 U IV bolus per hour until normalization of glucose levels; afterwards, he was switched to a bimodal schedule of SC insulin therapy: 5 U of Crystalline preprandially and 14 U of NPH at bedtime. The first dose of NPH caused immediate hypersensitivity, with urticaria lesions, pruritus, and low fever. Thus, this presentation was omitted, and IV chlorpheniramine was used twice per day for treatment of the allergic reaction; he was discharged 3 days later with SC Crystalline as sole treatment, at a dosage of 15 U 30 minutes prior to each meal.

Two weeks later, in November 2012, the patient returns to the local ER with moderate dehydration, multiple emesis, abdominal pain, and polyuria. He was started on fluids, empirical antibiotic therapy, and IV Crystalline boluses, and after normalization of glucose levels, he was administered 32 U of Glargine SC which induced an immediate anaphylactoid reaction with angioedema, wheezing, shortness of breath, and pruritic rash. He was treated with IV hydrocortisone (1 g) and chlorpheniramine (10 mg), and the allergic symptoms remitted after 36 hours. He was discharged again 1 week later with SC Lispro (Humalog, 18 U 15 minutes prior to every meal) insulin as treatment. During the first weeks of December 2012, the mother started to notice small urticaria lesions on her son's legs and torso 2 hours after insulin injection, which disappeared with the use of common oral antihistamines such as Loratadine. However, she became aware that the dosage for insulin appeared insufficient and had to be increased progressively in order to try and achieve glycemic control, but skin lesions were spreading further around his body.

During the final week of December 2012, the patient was referred to the University Hospital in the city of Maracaibo due to hyperglycemia, ketoacidosis, profound weight loss (20 kilograms since October 2012), muscular wasting, and general malaise. During physical examination the following was observed: profuse diaphoresis, pale skin, abdominal distention and flatulence, no signs of neurological focalization, or disorientation; respiratory rate: 22 bpm, heart rate: 121 bpm, blood pressure: 100/60 mmHg, and temperature: 36.8°C; regarding anthropometric measurements, height: 183 cm, weight: 47 kg, arm span: 185 cm, and BMI: 14.07 kg/m^2^. He was admitted with a diagnosis of diabetic ketoacidosis and treated with both 100 U of Crystalline insulin diluted in 500 cc of 0.9% saline solution at a rate of 5 UI/kg/h and chlorpheniramine (10 mg) STAT intravenously. After 4 hours, glucose levels normalized and no signs of allergy were observed. However, the following day the patient develops symptomatic hypoglycemia and insulin treatment is modified. A bimodal scheme is installed with 5 U SC of Crystalline insulin preprandially, maintaining the dose of IV chlorpheniramine every 12 hours. Forty-eight hours later, no skin lesions resembling hypersensitivity were observed, but glycemic control was suboptimal, with reports of preprandial glycemia between 250–480 mg/dL at noon. Insulin therapy was then further modified to include a 15 U prebreakfast bolus of Crystalline insulin, 5 U before lunch and before dinner, and a final 10 U bedtime bolus of NPH insulin, both SC. However, this schedule was omitted immediately because the patient developed angioedema with the first dose of NPH.

**Figure 3:**
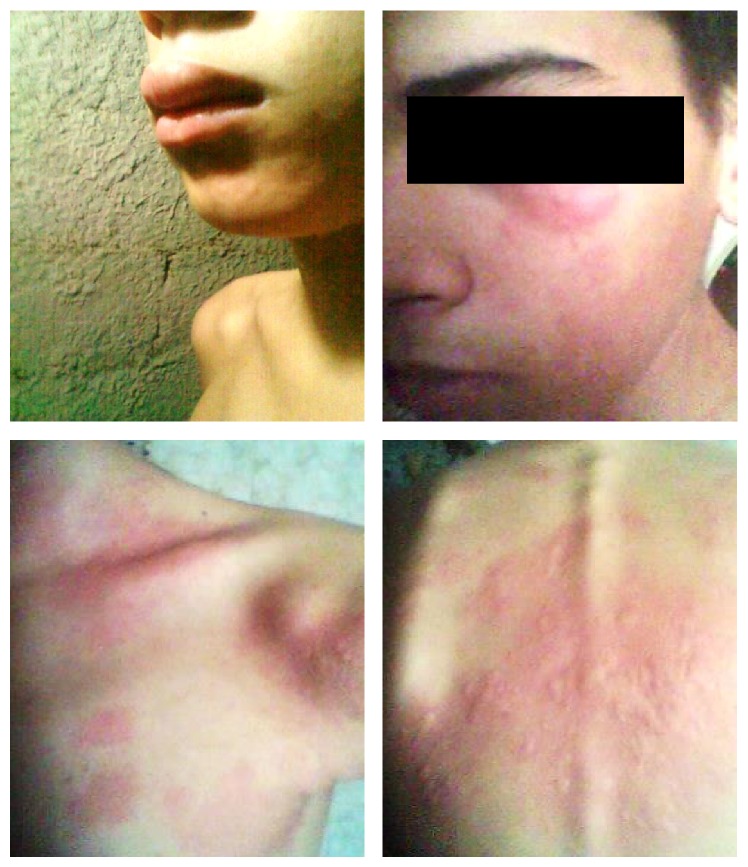
Allergic reaction to Detemir. Note distribution of wheals and flares, as well as angioedema of lips and eyelids.

It is after this last hypersensitivity episode that our Endocrinology and Immunology Departments were asked to evaluate the case. A Prick Test with different types of insulin was performed in order to determine which preparation would be most appropriate for the patient. All commercially available presentations were used with the exception of Aspart (NovoRapid) insulin (which was not available in the city at the time). The test was positive for NPH and Lispro, while it was negative for Glargine, Detemir (Levemir), and glulisine (Apidra). Insulin therapy was then started with a basal-bolus scheme: glulisine 10 U SC preprandially and Detemir 20 U SC at bedtime, accompanied with 10 mg of Ebastine p.o. every 8 hours, and 10 mg of Prednisone p.o. once per day. Normalization of glycemic values was obtained with this scheme with no allergic skin lesions, so the patient was discharged. During hospitalization, several laboratory panels were undertaken to further investigate insulin allergy and to exclude involvement of autoimmunity in other glands, which would suggest diagnosis of APS (Table 3); these explorations returned positive for autoimmune gastritis/pernicious anemia. Additionally, he was evaluated for Marfan's syndrome due to his height and arm span measurements; however ophthalmologic, radiologic, and cardiologic testing ruled out this diagnosis. One week after discharge, the patient attends emergency services again due to pruriginous papules in the neck, thorax, and limbs that appeared approximately 45 minutes after the Detemir injections (Figure 3), and one event during dinner-time injection associated with intense pruritus with glulisine dosage. In light of these findings, a desensitization protocol was conceived and applied.

The desensitization protocol is described in Table 4. The purpose of this therapy is to induce skin anergy by multiple and increasing dosages of insulin until a given dosage of the medication is tolerated by cutaneous immunocytes. Since the patient tolerated Crystalline via SC, we decided to use Glargine insulin because of local availability and economic factors. The protocol included premedication with 10 mg of Ebastine and 60 mg of Prednisone 30 minutes before the first dose. Glargine was administered intradermally in the abdominal region every 20 minutes for 5 days; see Table 3 for dosages. The goal of the procedure was to induce tolerance to dosages of 12 U Glargine, given twice almost simultaneously to achieve a total of 24 U of this insulin per day in two different body regions. The dose of Prednisone was then lowered to 30 mg during days 2 and 3 and suspended on day 4; glucocorticoid therapy lasted a total of 4 days. The patient was discharged seven days after admission. One year later, he is stable and without any flaring event, studying second semester at community college. Currently assisting to the outpatient consults every 4 months.

## 3. Discussion

Insulin allergy is a rare and complex complication of insulin therapy in diabetic patients, with a current estimated prevalence of approximately 2.4% [8], depending on case reports in type 1 and type 2 diabetes mellitus patients. The importance of insulin allergy relies on its fundamental role as a lifelong treatment. Several cases have been documented on diabetic patients, allergic to Glargine [32, 33], Detemir [34, 35], Crystalline [14, 36], NPH [14, 37], Aspart [14], Lispro [33], all available insulins [8, 12, 15, 38], and even components of such medications such as metacresol [24] and protamine [10, 19, 33], with or without presence of beta-lactam antibiotic allergy [12]. Clinical presentation varies, from local cutaneous lesions to anaphylactic shock, either IgE- or IgG-mediated [39]; see Figure 1. Type I allergy reactions are IgE-dependent, induced by the insulin molecule or other components, activating the allergy-related pathway. Nevertheless, IgG-mediated insulin allergy has also been reported [15, 40].

Heinzerling et al. [39] proposed a diagnostic flowchart, suggesting manifestations related to the acute presentation (urticaria, rash, angioedema, dyspnea, nausea, diarrhea, and cardiovascular manifestations) are likely to be IgE-mediated and suggest the need for skin prick testing and assessment of IgE-insulin-specific titers. On the other hand, type IV late signs such as induration and erythema at injection site suggest IgG-mediated allergy, relating to specific antiinsulin and anticomponent IgG titers. In the present case, we assessed a young man who had acute presentation symptoms when treated with Glargine, NPH, Lispro, Detemir, and Glulisine, which evolved over a period of approximately 3 months. When insulin allergy is diagnosed, the general recommendation is to change to another insulin preparation and observe tolerance, which in this case failed in each attempt, demonstrated by cutaneous manifestations and severe inadequate glycemic control. An important limitation in this case, was the lack of quantification of insulin-specific IgE/IgG antibodies, as these assay kits are not available in our country. It is noteworthy to comment about the results of the prick test. Glargine was negative in this test, even though he had experienced hypersensitivity to this type of insulin when injected with 32 U. We propose that negativity for this insulin might be due to (a) very high dosage used in this primary treatment which might have caused an adverse drug reaction, or (b) Glargine allergy-induction needs higher doses than the one used in the prick test. Previous case reports have suggested that insulin-related allergy depends on dosage, route of administration, and type of insulin and these account for discrepancies observed between allergenic crisis and skin testing [14, 39, 41].

Given the escalating insulin allergy events observed, a desensitization protocol was planned with Glargine, a long-lasting insulin formulation that is easily available in the country. The purpose of this protocol is to control local forms of insulin allergy, inducing anergy in skin immunocytes [26]. Other protocols have used Glargine [33, 39, 41] and other insulin analogs, such as Aspart [8, 24] for their desensitization therapy, either using continuous infusion [24, 40, 41] or intradermal injections, all of them achieving immunological control and metabolic improvement.

To our knowledge, this is the first documented case of insulin allergy in type 4 APS. Both clinical entities share similar immunogenetics: insulin immunogenicity is intimately related to T1DM pathogenesis [42] and might share HLA haplotypes with APS. Although HLA-B15-DR4 boasts an important association with hyperimmune manifestations [43], insulin-allergic subjects exhibit a greater prevalence of HLA-Bw44, HLA-DR7, and HLA-A2 and their combinations, with a RR of 20.6 for developing an allergy/immune reaction to insulin [44] and the presence of a specific immune response gene for insulin [43]. On the other hand, APS encompasses a constellation of immunogenic diseases (Table 2) whose presentation and severity rely on the convergence of predisposing and protective HLA haplotypes. In regards to the present case (APS-4: vitiligo + T1DM + autoimmune gastritis/pernicious anemia), vitiligo has been associated with cytotoxic T lymphocyte-mediated melanocyte destruction in patients positive for HLA-A2 [45], HLA-DRB1∗07-DQB1∗02 [46], and HLA-B∗44:03/DRB1∗07:01 [47]. As for T1DM, several alleles have been proposed, and although they appear to be highly ethnicity-specific, HLA-A2 [48], HLA-DR4-DQ8, and DR3-DQ2 [49] are the most prevalent worldwide. Lastly, autoimmune gastritis has been described to be mainly associated with DRB1∗1101/DQA1∗0505 [50] and HLA-DRB1∗04/DQB1∗03 [51], whereas pernicious anemia is associated with higher prevalence of HLA-D/DR3 and HLA-DR5 [52].

Cases of APS type 4 have been published previously, although with different combinations when compared to ours. Krysiak et al. [53] published a case concerning primary hypoparathyroidism and T1DM, associated with positive anti-transglutaminase and anti-parietal cell antibodies, while Hsu et al. [54] reported a peculiar case of T1DM, anti-GAD-related dystonia, vitiligo,* alopecia areata,* and* myasthenia gravis*. The present case is characterized by the primary development of vitiligo, followed by T1DM, and the detection of autoimmune gastritis/pernicious anemia while investigating insulin allergy, making this case one of a kind.

## 4. Conclusions

Insulin allergy is considered a rare complication during insulin therapy, yet it should always be suspected in patients with nonspecific cutaneous and systemic symptoms after insulin injections, where appropriate insulin-specific IgE assays should be carried out. The presence of two or more autoimmune-related diseases must prompt further evaluation for other affected organs and classification of the patient. Ultimately, this scenario begs the question: is insulin allergy a sign of an underlying major autoimmune disease, such as APS and nonconventional organ-specific autoimmunity? Further immunogenetic and pathophysiologic studies may help clarify this enigma, especially in the case of this pair of disorders, which seem to be more prevalent in current medical practice.

## Figures and Tables

**Table 1:** Insulin analogues and recombinant variations, structure, and related immunogenic reactions.

Immunogenic molecules	Reaction type	References
Regular human insulin		
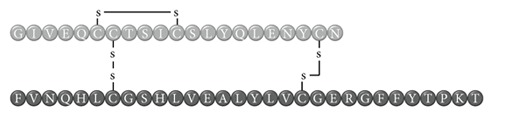		
Insulin	I	[8, 10–15]
	III	[15]

Crystalline insulin		
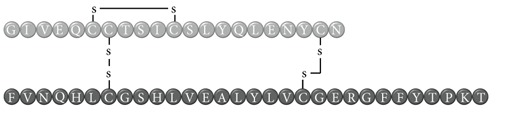		
Insulin	I	[8, 16, 17]

Porcine insulin		
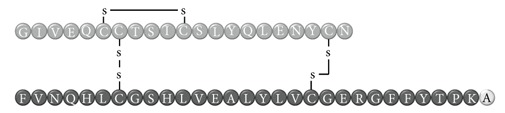		
Insulin	I	[8, 16]

Bovine insulin		
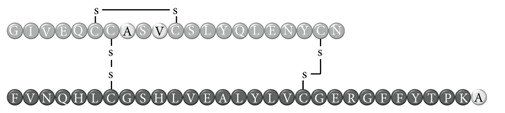		
Insulin	I	[8, 16]

Neutral Protamine Hagedorn (NPH) insulin		
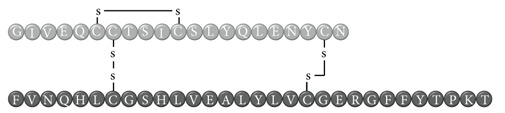		
Insulin	I	[8, 10, 11, 17]
	IV	[18]
Protamine	I	[8, 10, 19]

Lispro insulin (Humalog)		
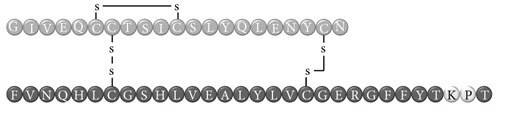		
Insulin	I	[8, 12, 15]
	III	[15]

Aspart insulin (NovoLog)		
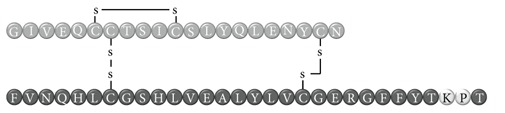		
Insulin	I	[8, 16, 20]
Glargine insulin (Lantus)		
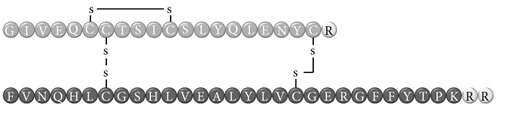		
Insulin	I	[11, 12, 21]
	III	[14]

Detemir insulin (Levemir)		
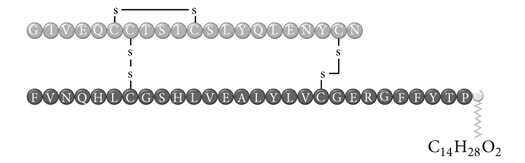		
Insulin	I	[12, 22]
	III	[14, 23]
	IV	[22]
Metacresol	I	[24]

**Table 2:** Classification of autoimmune polyglandular syndromes (APS).

Category	Subtypes	Criteria
**APS-1**	—	Two or more from the following (i) Addison's disease (ii) Chronic candidiasis (iii) Hypoparathyroidism Associated conditions (i) Alopecia (ii) Autoimmune gastritis/pernicious anemia (iii) Type 1 diabetes (iv) Vitiligo (v) Autoimmune thyroid disease (vi) Chronic hepatitis (vii) Autoimmune-related gonadal failure

**APS-2**	—	Addison's disease plus any of the following (i) Type 1 diabetes (ii) Autoimmune thyroid disease

**APS-3**	APS-3A APS-3B APS-3C APS-3D	Autoimmune thyroid disease plus: type 1 diabetes with/without any other endocrine organ involvement Autoimmune thyroid disease plus: autoimmune gastrohepatic disease (inflammatory bowel syndrome, pernicious anemia, autoimmune gastritis, and primary biliary cirrhosis) Autoimmune thyroid disease plus: skin autoimmune disease (vitiligo with/without alopecia areata) with/without nervous system autoimmune disease (miastenia gravis, multiple sclerosis) Autoimmune thyroid disease plus: rheumatological autoimmune disease (systemic and discoid lupus, rheumatoid arthritis, Sjögren syndrome, systemic sclerosis, vasculitis, and antiphospholipid syndrome) with/without hematological disease

**APS-4**	—	Any other combination of specific organ and nonorgan specific autoimmune diseases

**Table 3:** Complementary laboratory workup.

	Results	Reference values
Immunoglobulin E (IgE)	140.60 IU/mL	0–150 IU/mL
Immunoglobulin A (IgA)	1 g/L	0.60–3.09 g/liter
Immunoglobulin M (IgM)	245 mg/dL	40–250 mg/dL
Immunoglobulin G (IgG)	1529 mg/dL	710–1520 mg/dL
Fasting C peptide	0.14 ng/mL	0.9–7.1 ng/mL
Cortisol (AM)	42.5	30–150 ng/mL
Free triiodothyronine (FT3)	1.9 pg/mL	1.4–4.2 pg/mL
Free thyroxine (FT4)	1.1	0.89–1.76 ng/mL
Thyroid stimulating hormone (TSH)	0.945 *μ*IU/mL	0.3–4.00 mUI/mL
Anti-thyroglobulin antibody	7.0 IU/mL	<10 IU/mL
Anti-thyroperoxidase antibody	10.1 IU/mL	<30 IU/mL
Parietal cell autoantibody	Positive	
Intrinsic factor autoantibody	Positive	
Anti-gliadin antibodies	Negative	
Anti-transglutaminase antibodies	Negative	
Anti-*Saccharomyces* antibodies	Negative	

**Table 4:** Desensitization protocol using Glargine insulin.

Day	Number of dosages^*^	Accumulated dose (IU)	Total dosage per day (IU)	Local reaction (cm) ^¶^
1	1	0,001	0,001	1
1	2	0,01	0,011	0
1	3	0,1	0,111	1,5
1	4	1	1,111	0
1	5	2	3,111	0
2	6	0,1	0,1	1
2	7	1	1,1	0
2	8	2	3,1	0
2	9	3	6,1	0
3	10	2	2	0
3	11	3	5	0
3	12	4	9	0
4	13	12	12	0
5	14	12	12	0
5	15°	12	24	0

^*^Time between injections: 20 minutes.

°Administrated almost simultaneously with dosage number 14, in a different corporal region.

^¶^Diameter of the flare.
